# Supplementary material for: Radiation-Induced Metabolic Shifts in the Hepatic Parenchyma: Findings from 18F-FDG PET Imaging and Tissue NMR Metabolomics in a Mouse Model for Hepatocellular Carcinoma
Source: Molecules. 2021 Apr 28;26(9):2573. doi: 10.3390/molecules26092573 (PMC8125521; doi:10.3390/molecules26092573)
Supplement: Supplementary file 1 [file molecules-26-02573-s001.zip › molecules-1195756-supplementary.pdf]

Table S1. Sequences of primers used for quantitative PCR reactions.

Gene name	Primer sequence (5′-3′)	Product size (bp)
β-actin F	ACCCTAAGGCCAACCGTGAA	193
β-actin R	ATGGCGTGAGGGAGAGCATAG	
IL-18 F	CAGGCCTGACATCTTCTGCAA	105
IL-18 R	TCTGACATGGCAGCCATTGT	
HIF-1α F	TCCATGTGACCATGAGGAAA	160
HIF-1α R	CTTCCACGTTGCTGACTTGA	
IL-1β F	TGAAGCAGCTATGGCAACTG	183
IL-1β R	TTGTTGATGT GCTGCTGTGA	
IL-6 F	AGTTGCCTTCTTGGGACTGA	159
IL-6 R	TCCACGATTTCCCAGAGAAC	
PCK1 F	CTGCATAACGGTCTGGACTTC	159
PCK1 R	CAGCAACTGCCCGTACTCC	
G6pc F	CGACTCGCTATCTCCAAGTGA	173
G6pc R	GTTGAACCAGTCTCCGACCA	
Fbp1 F	CACCGCGATCAAAGCCATCT	161
Fbp1 R	AGGTAGCGTAGGACGACTTCA	
PC F	CTGAAGTTCCAAACAGTTCGAGG	162
PC R	CGCACGAAACACTCGGATG	

Figure S1. The SUVmean and SUVmax values measured in the left hepatic parenchyma on the first and third post-irradiation days. There was no significant increased FDG uptake between RT (n=6) group and no-RT (n=6) group in post-irradiation days.

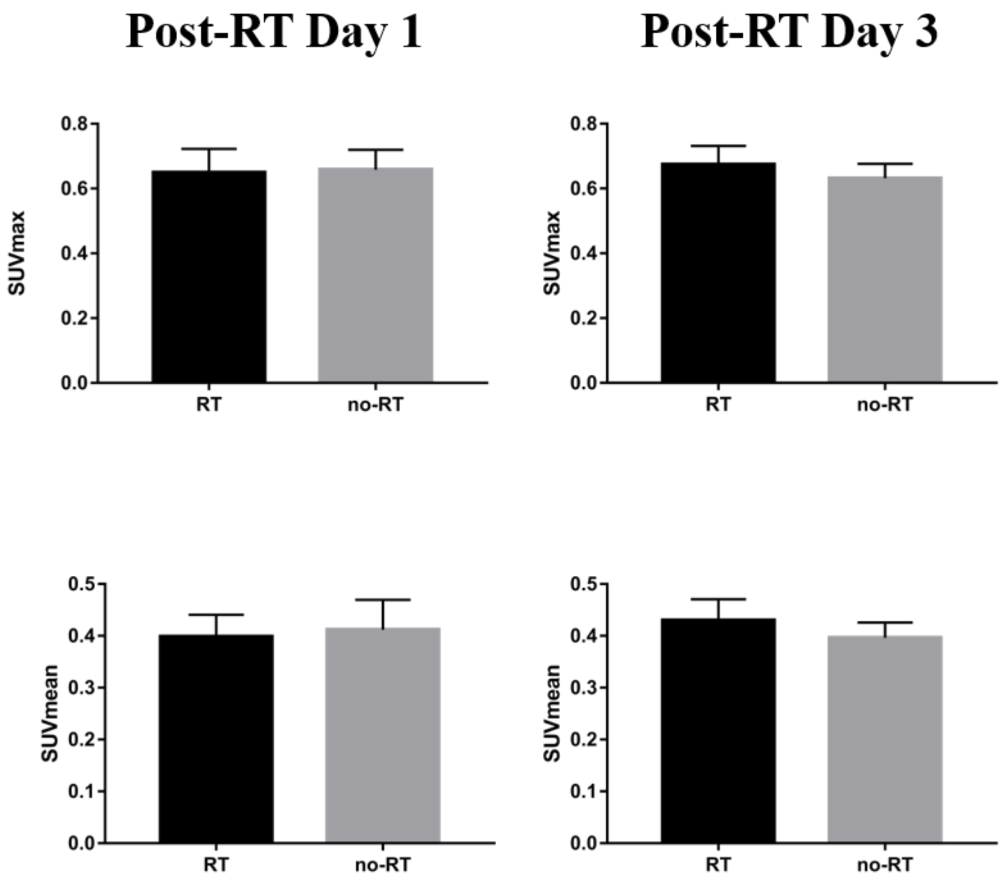

Figure S2. The comparison of FDG uptake expressed as the ratio of right to left lobes in tumor-free animals with and without irradiation after 3 day RT. In experiments conducted in tumor-free animals, the right-to-left ratio of ^{18}F-FDG uptake in the liver parenchyma measured on post-RT day 3 was 1.13-fold higher in the RT (n=4) group compared with the no-RT (n=4) group (1.11 ± 0.10 *versus* 0.98 ± 0.03, respectively, $p<0.05$).

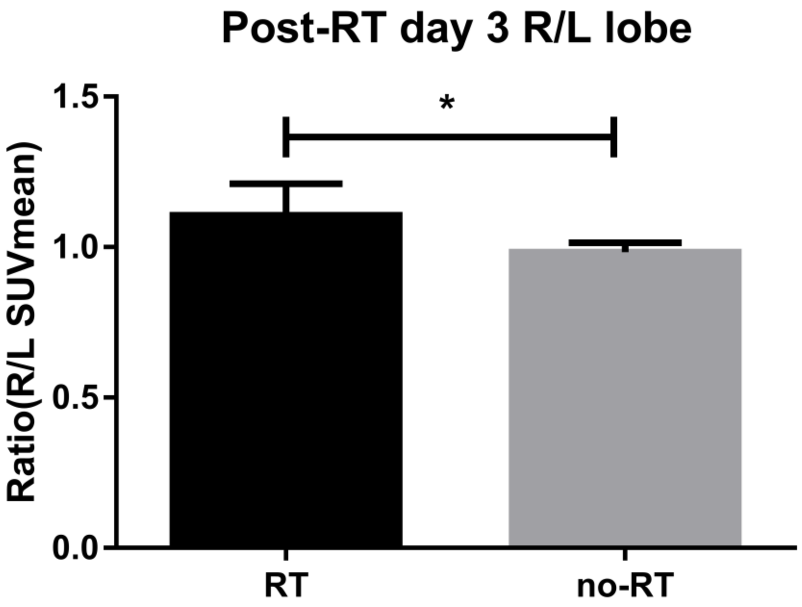

Figure S3. Lymphocytes and macrophages subpopulations within liver parenchyma identified using CD8, F4/80 and CD68 immunohistochemical staining. Representative section of right liver parenchyma stained with (a) CD8, F4/80, and CD68 on post-RT day 1 in the RT group, compared to the no-RT group. (b) CD8, F4/80, and CD68 on post-RT day 3 in the RT group, compared to the no-RT group. Nonetheless, there was no significant expansion of CD8+ T cells as well as of the F4/80+ or CD68+ macrophage populations in liver tissues on post-RT days 1 and 3

a. Post-RT day 1

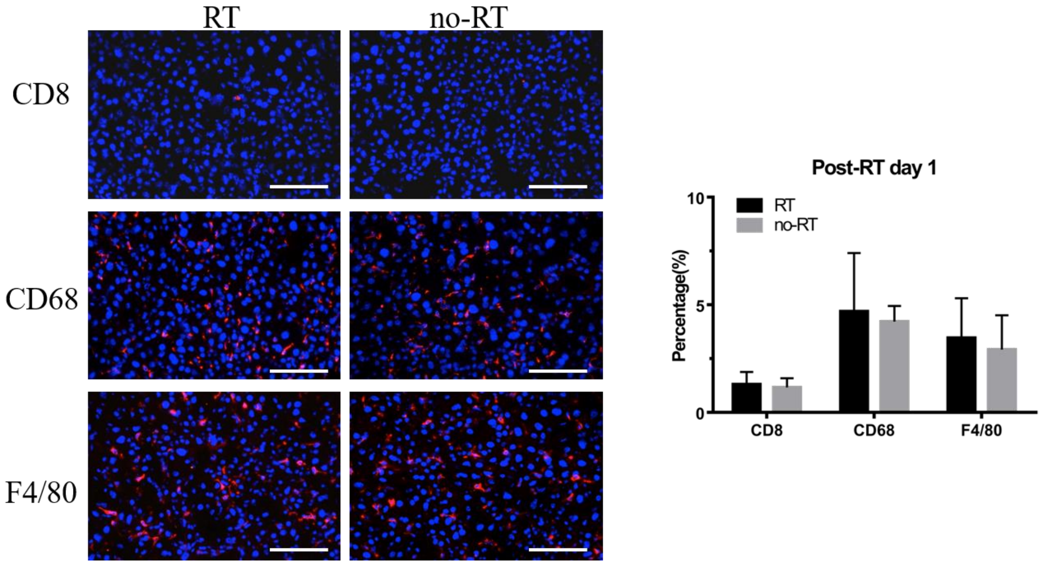

b. Post-RT day 3

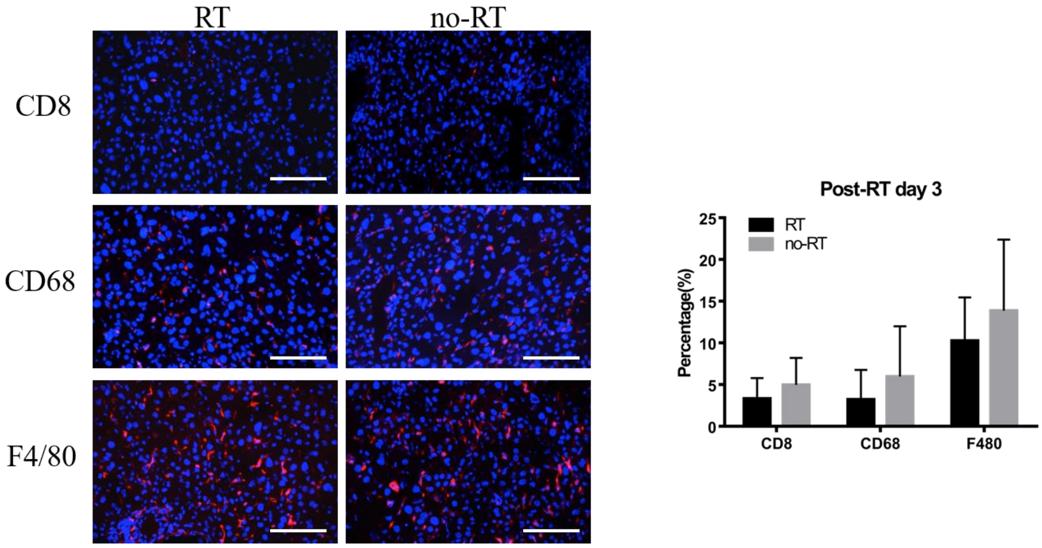

Figure S4. Expression levels of genes encoding for pro-inflammatory cytokines in the RT and no-RT groups on the first and third post-irradiation days (left lobe of the liver). On the first post-irradiation day, the expression levels of the IL-18 and IL-6 genes (expressed as fold-change) were significantly increased in the RT group – but not in the no-RT group. On the third post-irradiation day, the expression levels of the IL-1β, HIF-1α, and IL-6 genes (expressed as fold-change) were significantly increased in the RT group – but not in the no-RT group.

Post-RT day 1

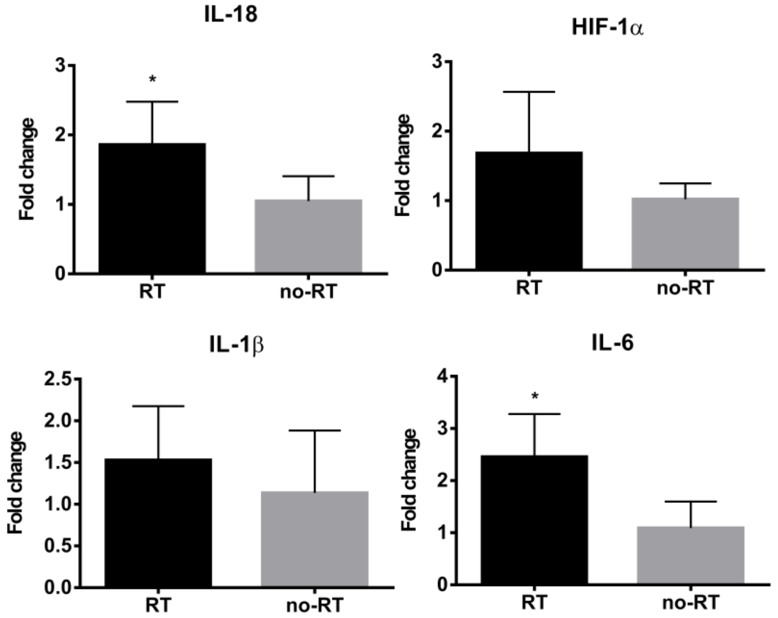

Post-RT day 3

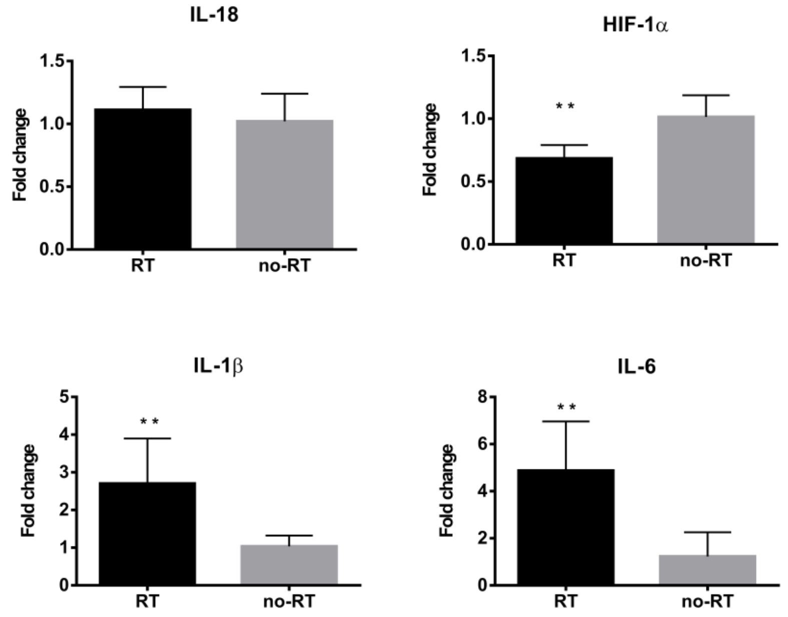

Figure S5. The extent of liver damage was assessed by measuring serum levels of AST, ALT, and albumin on the first and third post-irradiation days. Compared with control animals (n=3) , the presence of experimental tumors resulted in increased ALT (36.4 ± 11.00 *versus* 52.67 ± 8.08 IU/L, respectively, p = 0.021) and albumin levels. However, ALT, AST and albumin levels did not show significant differences in the RT (n=3) and no-RT (n=3) groups on post-RT neither day 1 nor day 3.

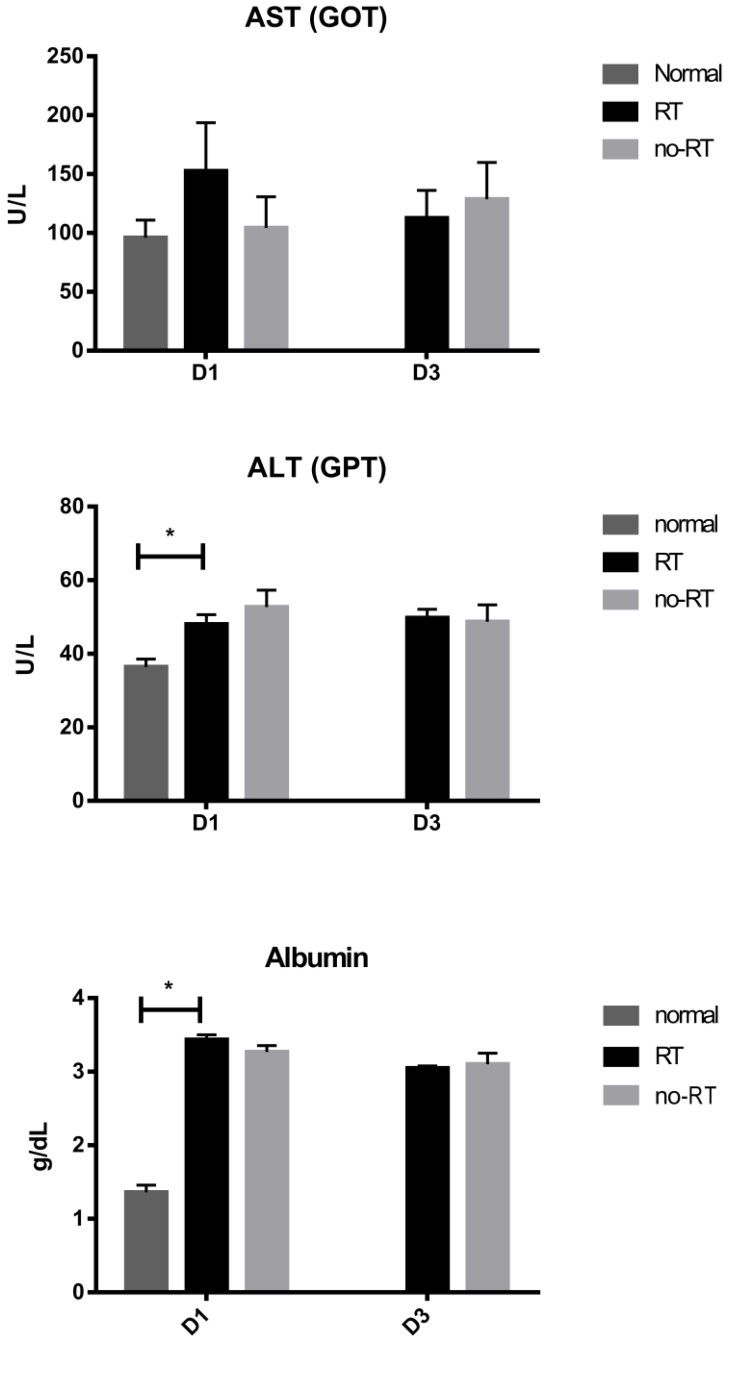

Table S2. Predicted (MetaboAnalyst 3.0) alterations in metabolic pathways in the right and left lobes of the liver on the first and third post-irradiation days

Region	*Metabolic pathway*	*P*
	**Post-RT day 1**	
Right lobe	Starch and sucrose metabolism	<0.001
	Galactose metabolism	<0.001
	Neomycin, kanamycin, and gentamicin biosynthesis	0.005
Left lobe	Galactose metabolism	0.008
	Alanine, aspartate, and glutamate metabolism	0.009
	Neomycin, kanamycin, and gentamicin biosynthesis	0.01
	**Post-RT day 3**	
Right lobe	Glycine, serine, and threonine metabolism	<0.001
	Arginine and proline metabolism	0.003
	Alanine, aspartate, and glutamate metabolism	0.02
	Glyoxylate and dicarboxylate metabolism	0.025
	Nitrogen metabolism	0.047
	D-glutamine and D-glutamate metabolism	0.047
Left lobe	Glycine, serine, and threonine metabolism	<0.001
	Tricarboxylic acid cycle	0.003
	Alanine, aspartate, and glutamate metabolism	0.007
	Riboflavin metabolism	0.018
